# Positive Effects of Physical Activity on Insulin Signaling

**DOI:** 10.3390/cimb46060327

**Published:** 2024-05-30

**Authors:** Paulina Małkowska

**Affiliations:** Institute of Physical Culture Sciences, University of Szczecin, 71-065 Szczecin, Poland; paulina.malkowska@usz.edu.pl

**Keywords:** physical activity, insulin sensitivity, metabolic health, inflammation, insulin signaling, exercise interventions, type 2 diabetes, metabolic syndrome

## Abstract

Physical activity is integral to metabolic health, particularly in addressing insulin resistance and related disorders such as type 2 diabetes mellitus (T2DM). Studies consistently demonstrate a strong association between physical activity levels and insulin sensitivity. Regular exercise interventions were shown to significantly improve glycemic control, highlighting exercise as a recommended therapeutic strategy for reducing insulin resistance. Physical inactivity is closely linked to islet cell insufficiency, exacerbating insulin resistance through various pathways including ER stress, mitochondrial dysfunction, oxidative stress, and inflammation. Conversely, physical training and exercise preserve and restore islet function, enhancing peripheral insulin sensitivity. Exercise interventions stimulate β-cell proliferation through increased circulating levels of growth factors, further emphasizing its role in maintaining pancreatic health and glucose metabolism. Furthermore, sedentary lifestyles contribute to elevated oxidative stress levels and ceramide production, impairing insulin signaling and glucose metabolism. Regular exercise induces anti-inflammatory responses, enhances antioxidant defenses, and promotes mitochondrial function, thereby improving insulin sensitivity and metabolic efficiency. Encouraging individuals to adopt active lifestyles and engage in regular exercise is crucial for preventing and managing insulin resistance and related metabolic disorders, ultimately promoting overall health and well-being.

## 1. Introduction

Insulin, an endocrine peptide hormone, interacts with receptors on cell membranes in specific cells to coordinate a comprehensive anabolic reaction to the presence of nutrients [1]. When glucose levels in the bloodstream rise to a certain point after eating, insulin is released by pancreatic β-cells. Typically, insulin facilitates the uptake of carbohydrates in vital locations for storage and utilization, like adipose tissue and skeletal muscle, where they are converted into lipids alongside proteins [2]. While insulin receptors are found in many somatic cell types, their role in maintaining glucose levels is mainly associated with their direct impact on the skeletal muscle, liver, and white adipocytes. These tissues have specific roles in metabolic balance, necessitating insulin’s tissue-specific signaling pathways. For instance, in skeletal muscle, insulin enhances glucose uptake and storage by boosting glucose transport and glycogen synthesis. In the liver, insulin activates glycogen synthesis, increases lipogenic gene expression, and reduces gluconeogenic gene expression. In white adipose tissue (WAT), insulin suppresses lipolysis while promoting glucose transport and lipogenesis. Despite these varied effects, the initial components of insulin signal transduction are quite similar across insulin-sensitive cells. However, the diverse physiological responses to insulin in different cell types mainly arise from distinct downstream effectors [1].

Decreased responsiveness of tissues to insulin results in insulin resistance (IR), a multifaceted pathological state marked by diminished sensitivity. This condition impairs the ability to suppress glucose production and enhance peripheral glucose disposal, often leading to elevated insulin levels to stabilize blood sugar levels [3]. IR is typified by compromised insulin-mediated blood glucose regulation, hindered glucose utilization, abnormal lipid accumulation, and heightened lipid breakdown in adipocytes. This constellation of symptoms is often referred to as insulin resistance syndrome or metabolic syndrome. IR is associated with conditions such as obesity, type 2 diabetes mellitus (T2DM) and its complications, non-alcoholic fatty liver disease (NAFLD), cancer, cardiovascular disease, and various other metabolic disorders [4]. The condition is considered a key precursor to the development of T2DM, as prolonged insulin resistance can lead to pancreatic β cell dysfunction and eventual failure to compensate for insulin resistance with increased insulin secretion [5,6]. Beyond its role in glucose metabolism, insulin resistance also affects lipid and protein metabolism. In adipose tissue, insulin resistance promotes lipolysis (breakdown of fats), leading to increased release of free fatty acids into the bloodstream, which contributes to dyslipidemia and ectopic fat deposition in non-adipose tissues [7]. Moreover, insulin resistance is associated with systemic inflammation and oxidative stress, further exacerbating metabolic dysfunction and increasing the risk of cardiovascular complications [8].

Given its profound implications for metabolic health, understanding and addressing insulin resistance are of paramount importance in preventing and managing metabolic disorders. Lifestyle modifications, including regular physical activity, healthy dietary habits, weight management, and stress reduction, play a crucial role in improving insulin sensitivity and mitigating the adverse effects of insulin resistance on metabolic health [9,10,11].

**Figure 1:**
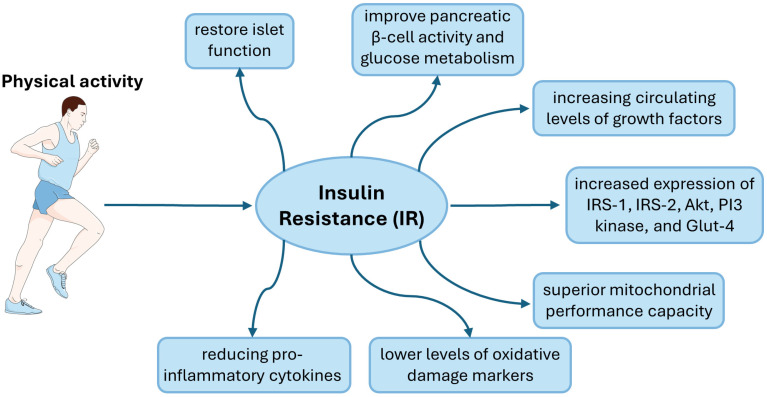
Graphic representation of the impact of physical activity in people with insulin resistance. This figure was partly generated using Servier Medical Art, provided by Servier, licensed under a Creative Commons Attribution 3.0 unported license.

Physical activity plays a pivotal role in modulating the biomarkers associated with insulin resistance by exerting beneficial effects on various physiological pathways involved in glucose and lipid metabolism (Figure 1). Regular physical activity enhances insulin sensitivity and improves glycemic control through multiple mechanisms, thereby mitigating the development and progression of insulin resistance-related disorders such as T2DM, obesity, and cardiovascular disease. As most studies indicate, insulin resistance is associated with excessive lipid accumulation in skeletal muscle and the liver, which is also linked to adipose tissue [12]. For this reason, this review will focus on the insulin signaling pathway in skeletal muscle, liver and adipose tissue.

## 2. Insulin Signaling Pathway

Insulin exerts its various physiological effects by binding to the insulin receptor (INSR) situated on the cell membrane of target cells. The INSR is a complex receptor composed of two extracellular α subunits, responsible for insulin binding, and two membrane-spanning β subunits, each containing a tyrosine kinase domain [13]. Among the two isoforms of INSR, isoform B is more specific for insulin and is predominantly expressed in mature liver, muscle, and WAT, mediating the majority of insulin’s metabolic effects. In contrast, isoform A, prevalent during fetal development due to its high affinity for IGF-2, differs by the exclusion of exon 11 [14]. The INSR possesses two insulin binding sites but exhibits negative cooperativity, whereby insulin binding at one site decreases the affinity for insulin binding at the other site. Thus, evidence suggests that one insulin molecule activates one INSR under physiological conditions [15]. Upon insulin binding, the β-subunit undergoes a conformational change, leading to autophosphorylation of tyrosine residues and recruitment of various adaptor proteins, such as insulin receptor substrate (IRS), growth factor receptor-bound protein-2 (GRB-2), GRB-10, SHC-transforming protein (SHC), and SH2B adapter protein-2 (SH2B-2) [16].

**Figure 2:**
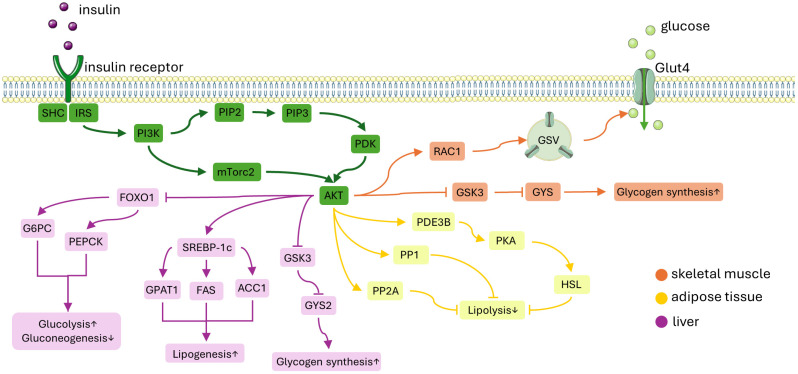
A simplified diagram of the insulin signaling pathway. Insulin attaches to the insulin receptor which activates IRS-1. This activation leads to the recruitment of PI3K and subsequently activates Akt. ORANGE PATH: In skeletal muscle, Akt facilitates glucose uptake by moving GSVs to the plasma membrane. This process involves the activation of the GTP-bound form of RAC1. Additionally, insulin promotes glycogen synthesis by inhibiting GSK3, which activates GYS, and by inactivating glycogen phosphorylase through the dephosphorylation of phosphorylase kinase. YELLOW PATH: In adipose tissue, insulin inhibits lipolysis by decreasing the availability of gluconeogenic substrates. This suppression is thought to be mediated by PDE3B, PP1, and PP2A. PURPLE PATH: In the liver, Akt reduces gluconeogenesis by inhibiting the expression of gluconeogenic genes mediated by FOXO1. Insulin enhances hepatic glycogen synthesis by regulating GYS2 and GSK3. Moreover, insulin promotes lipogenesis by up-regulating SREBP-1c. Abbreviations: IRS-1, insulin receptor substrate-1; PI3K, phosphatidylinositol-3-OH kinase; GSVs, glucose transporter type 4 (GLUT4) storage vesicles; RAC1, Ras-related C3 botulinum toxin substrate 1; GYS, glycogen synthease; PDE3B, phosphodiesterase 3B; PP1, protein phosphatase 1; PP2A, protein phosphatase-2A; FOXO1, forkhead box O1; GSK3, glycogen synthase kinase 3; SREBP-1c, sterol regulatory element-binding protein 1c; PIP2, phosphatidylinositol-4,5-bisphosphate; PIP3, phosphatidylinositol-3,4,5-trisphosphate; mTORC1, mechanistic target of rapamycin complex 1; G6PC, glucose-6-phosphatase; PEPCK, phosphoenolpyruvate carboxykinase 1; GPAT1, glycerol-3-phosphate acyltransferase 1; ACC, acetyl-CoA carboxylase; FAS, fatty acid synthase. This figure was partly generated using Servier Medical Art, provided by Servier, licensed under a Creative Commons Attribution 3.0 unported license.

Insulin’s impact on glucose and lipid metabolism primarily occurs through a series of molecular events initiated by the insulin receptor tyrosine kinase (IRTK). This process involves the phosphorylation of insulin receptor substrate (IRS), which subsequently attracts phosphatidylinositol-3-OH kinase (PI3K). PI3K then triggers the conversion of phosphatidylinositol-4,5-bisphosphate (PIP2) into phosphatidylinositol-3,4,5-trisphosphate (PIP3). Akt, once recruited to the cell membrane by PIP3, becomes activated through phosphorylation by both 3-phosphoinositide-dependent kinase-1 (PDK1) and mechanistic target of rapamycin complex 2 (mTORC2) [17]. This activated Akt proceeds to phosphorylate a variety of downstream substrates in metabolic tissues such as skeletal muscle, liver, and adipose tissue (Figure 2). These phosphorylation events initiate insulin-induced mechanisms that promote the retention of nutrients within these tissues.

### 2.1. Insulin Signaling Pathway in Skeletal Muscle

Skeletal muscle is a highly energetically demanding tissue, requiring substantial resources to function efficiently. The energy stored within muscle cells is predominantly reserved for future needs. However, there is an exception with the three-carbon molecules, such as lactate and alanine. Lactate is formed by the reduction of pyruvate by NADH in a reaction catalyzed by lactate dehydrogenase as the end product of anaerobic glycolysis. Alanine can be formed from pyruvate and glutamate catalyzed by alanine aminotransferase. These molecules are released from the skeletal muscle and are transported to the liver, serving as substrates for further metabolic processes [1]. In skeletal muscle, insulin signaling plays a crucial role in facilitating glucose uptake and promoting net glycogen synthesis. Upon insulin activation, there is a coordinated process that enhances glucose transport activity through the translocation and fusion of glucose transporter type 4 (GLUT4) storage vesicles (GSVs) to the plasma membrane within skeletal muscle. This process is mediated by the inactivation of AS160 (GTPase-activating protein [GAP] AKT substrate of 160 kDa, also known as TBC1D4) by Akt, which, in turn, activates small Rab GTPase proteins that control vesicle trafficking [18]. Additionally, insulin-induced Akt activation promotes the GTP-bound form of Ras-related C3 botulinum toxin substrate 1 (RAC1), facilitating GLUT4 translocation by inducing the reorganization of cortical actin [19,20,21]. RAC1, a protein involved in cellular signaling, has direct targets such as p21-associated kinase (PAK). Insulin enhances the activation of RAC1, promoting its GTP-bound form, which in turn stimulates the phosphorylation of PAK by alleviating PAK autoinhibition [19,20]. When RAC1 is specifically knocked out in muscle cells, there is a significant impairment in insulin-stimulated glucose uptake, even though AKT activation remains intact [22]. Conversely, when constitutively active RAC1 is overexpressed in muscle cells, it induces GLUT4 translocation, facilitating glucose uptake, even in the absence of insulin stimulation [23].

Furthermore, insulin regulates net glycogen synthesis in skeletal muscle by suppressing glycogenolysis and promoting glycogen synthesis. This regulation occurs through several mechanisms. Firstly, insulin signaling enhances the activity of glycogen synthase (GYS) by phosphorylating glycogen synthase kinase 3 (GSK3) via Akt activation. Secondly, insulin activates protein phosphatase 1 (PP1), promoting the dephosphorylation of GYS, thus further enhancing glycogen synthesis [24,25]. PP1 phosphatase activity serves multiple targets in various cellular pathways. However, its specificity towards glycogen synthase is mediated by four glycogen-targeted PP1 regulatory subunits [25]. These regulatory subunits contain binding domains for PP1, GYS, and glycogen, functioning as metabolic scaffolds [26]. Among these regulatory subunits, G_M_ exhibits the highest expression in skeletal muscle. Mice lacking G_M_ expression demonstrate reduced muscle glycogen stores [27,28,29]. Insulin promotes the recruitment of PP1 to glycogen molecules and enhances PP1 activity towards GYS. However, the precise molecular mechanisms underlying this action remain incompletely understood [26]. Conventionally, the combination of inactive glycogen synthase kinase 3 (GSK3) and active PP1 promotes the formation of active, dephosphorylated muscle glycogen synthase, thereby facilitating glycogen synthesis [30]. Additionally, insulin regulates glycogen phosphorylase activity by promoting the dephosphorylation of phosphorylase kinase. Collectively, these insulin-mediated processes promote glucose uptake and glycogen synthesis, contributing to the efficient storage of glucose within skeletal muscle cells [31].

### 2.2. Insulin Signaling Pathway in WAT

The primary physiological role of insulin in white adipose tissue is to inhibit lipolysis, which consequently suppresses hepatic glucose production (HGP) by reducing gluconeogenic substrates [32]. In vivo, white adipocytes exhibit high sensitivity to insulin [1]. Insulin’s ability to regulate plasma levels of non-esterified fatty acids (NEFAs) is essential for maintaining normal blood glucose levels. Inhibition of lipolysis, a process by which triglycerides are broken down into NEFAs and glycerol, represents a significant physiological function of insulin in WAT [33,34]. The precise mechanism behind insulin-induced suppression of lipolysis is not entirely elucidated, but evidence suggests that phosphodiesterase 3B (PDE3B) plays a role through decreased activity of cyclic adenosine monophosphate (cAMP)-dependent protein kinase A (PKA) [35]. PKA plays a crucial role in the regulation of WAT lipolysis by phosphorylating two key proteins: hormone-sensitive lipase (HSL) and perilipin (PLIN) [36]. HSL undergoes phosphorylation at three serine residues located at the COOH end, resulting in its translocation from the cytosol to the surface of lipid droplets [37,38]. The significance of HSL in the hormonal control of WAT lipolysis was underscored by the discovery of frame shift mutations in the human HSL gene. Individuals homozygous for this mutation lack HSL expression and exhibit severely impaired lipolysis regulation [39]. Perilipins, which encompass five isoforms with tissue-specific functions, are abundantly expressed [40]. PLIN1, predominantly found in white adipocytes, is phosphorylated by PKA at multiple serine residues [40,41]. While the precise functions of PLIN phosphorylation in lipolytic regulation are not fully elucidated, they are believed to involve several major mechanisms. Firstly, phosphorylation of PLIN reduces its affinity for the adipose triglyceride lipase (ATGL) cofactor CGI-58, facilitating CGI-58 binding to ATGL and thereby enhancing ATGL activity by approximately 20-fold [41]. Secondly, PLIN phosphorylation is crucial for the complete activation of HSL on the surface of lipid droplets [38]. Thirdly, PLIN phosphorylation was demonstrated to increase the surface area-to-volume ratio of lipid droplets by promoting the budding of lipid microbubbles [42]. PDE3B serves to degrade cAMP, thereby dampening prolipolytic PKA signaling towards HSL and PLIN. In Pde3b-deficient adipocytes, stimulated lipolysis remains unimpeded by insulin suppression, and Pde3b knockout mice exhibit impaired suppression of plasma NEFA levels during glucose tolerance tests [35].

Furthermore, protein phosphatase-1 (PP1) and protein phosphatase-2A (PP2A) seem to facilitate PI3K-dependent insulin-induced suppression of lipolysis by dephosphorylating lipolytic regulatory proteins [43,44]. PP2A emerges as the primary mediator responsible for the dephosphorylation of HSL, even in scenarios where PKA activity is undetectable [45]. Conversely, PP1 is identified as the primary perilipin phosphatase in adipocytes. In response to insulin, both the phosphorylation and activity of PP1 regulatory subunits are observed to increase [43].

Although insulin facilitates glucose transport by triggering phosphorylation of targets involved in vesicle tethering, docking, and fusion, its impact on whole-body glucose disposal is relatively modest [46]. Insulin also stimulates lipogenesis in white adipose tissue by activating sterol regulatory element-binding protein 1c (SREBP-1c), promoting the translocation of glucose or fatty acid transport proteins (FATPs), encouraging fatty acid esterification, and fostering adipogenesis through the transcription factor peroxisome proliferator-activated receptor-γ (PPARγ) [47,48].

### 2.3. Insulin Signaling Pathway in Liver

Insulin, produced by the pancreatic endocrine gland, is released into the portal vein. As a result, the liver is exposed to higher concentrations of insulin, typically two to three times greater than those found in the general circulation [49]. Insulin plays a crucial role in promoting the synthesis of all major classes of metabolic macromolecules, including glycogen, lipids, and proteins. Additionally, insulin rapidly and effectively reduces hepatic glucose production. This reduction in HGP is particularly significant because increased fasting concentrations of HGP and its insensitivity to insulin are characteristic features of type 2 diabetes. Therefore, measuring the suppression of HGP by insulin is a widely recognized physiological indicator of liver insulin sensitivity [1,49]. In the liver, insulin initiates a signaling cascade by activating INSR, which subsequently phosphorylates insulin receptor substrate 1 (IRS1) and IRS2. The different genetic disorders affecting IRS1 and IRS2 expression in the liver have not definitively outlined the distinct functions of any specific isoform [1]. This leads to the activation of Akt2, a pivotal regulator of hepatic glucose metabolism. Akt2 exerts several effects that collectively reduce HGP, promote glycogen synthesis, and enhance lipogenesis [50].

The primary function of insulin signaling in the liver is to decrease HGP by inhibiting gluconeogenesis. This occurs through Akt-induced phosphorylation of forkhead box O1 (FOXO1), which results in the exclusion of FOXO1 from the nucleus. As a consequence, FOXO1 is unable to activate the transcription of gluconeogenic genes, such as glucose-6-phosphatase (G6PC) and phosphoenolpyruvate carboxylase (PEPCK) [50,51]. Active FOXO1 additionally associates with the co-repressor SIN3A to diminish the expression of glucokinase, thereby enhancing the preference for glucose export [52]. Additionally, insulin suppresses hepatic gluconeogenesis by inhibiting adipocyte lipolysis, which reduces the availability of gluconeogenic substrates in the liver [33].

In addition to inhibiting gluconeogenesis, insulin promotes hepatic glycogen synthesis by regulating glycogen synthase (especially GYS2 in the liver) and glycogen phosphorylase. This regulation is mediated through glycogen synthase kinase 3 (GSK3) and PP1, similar to the mechanisms observed in skeletal muscle [53].

Furthermore, insulin activates lipid synthesis (lipogenesis) in the liver by up-regulating SREBP-1c, a master transcriptional regulator of hepatic de novo lipogenesis. Subsequently, SREBP-1c enhances the transcription of several lipogenic genes, including acetyl-CoA carboxylase 1 (ACC1), fatty acid synthase (FAS), and glycerol-3-phosphate acyltransferase 1 (GPAT1). This results in increased synthesis of fatty acids and triglycerides, contributing to lipid storage and energy metabolism in the liver [54,55]. Liver-specific overexpression of SREBP-1c alone is adequate to induce hepatic steatosis [56]. Insulin primarily influences SREBP-1c by enhancing its transcription, but it also facilitates SREBP-1c cleavage and its subsequent translocation into the nucleus, which are the conventional mechanisms of SREBP activation [54,57]. These actions of insulin can be hindered by inhibiting PI3K, AKT, or mTORC1, indicating that these kinases serve as upstream regulators of SREBP-1c [58].

## 3. Mechanisms of Insulin Resistance

**Figure 3:**
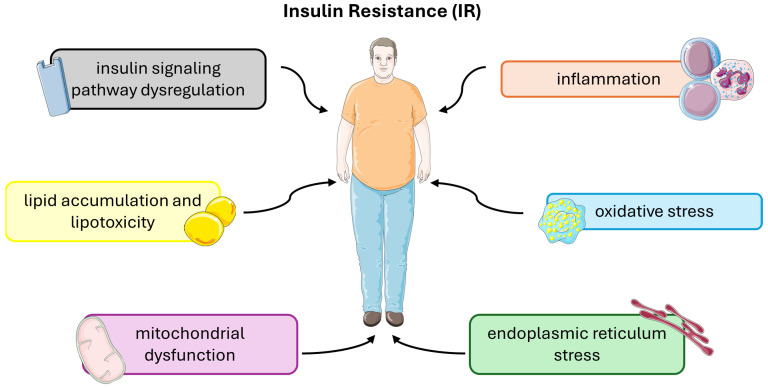
Molecular and cellular mechanisms that contribute to the development and progression of insulin resistance. This figure was partly generated using Servier Medical Art, provided by Servier, licensed under a Creative Commons Attribution 3.0 unported license.

Insulin resistance (IR) is a complex metabolic disorder characterized by impaired cellular responses to insulin, primarily affecting skeletal muscle, liver, and adipose tissue. Several underlying molecular and cellular mechanisms contribute to the development and progression of insulin resistance (Figure 3).

### 3.1. Insulin Signaling Pathway Dysregulation

Insulin receptors (INSRs), functioning as tyrosine kinases, have a specific affinity for insulin and are pivotal in regulating insulin-dependent glucose homeostasis and cellular growth [59,60]. Impaired binding of INSR primarily refers to reduced affinity or numbers of receptors on the cell membrane, or structural abnormalities affecting insulin’s interaction with the receptor [61]. The insulin receptor substrate protein serves as a critical node in the insulin signaling network and is closely linked to the development of insulin resistance. Molecularly, crosstalk between downstream nucleotide-binding oligomerization domain (NOD) 1 and the insulin receptor pathway can hinder insulin signaling by impeding the action of insulin receptor substrates [62].

Recent research indicates that insulin-stimulated kinases play a role in feedback phosphorylation of serine/threonine residues in IRS, contributing to the desensitization of proximal insulin signaling, which is significant in insulin resistance development [63]. For instance, a double-stranded RNA-dependent protein kinase (PKR) was found to elevate inhibitory IRS1 phosphorylation and IRS2 expression in liver and muscle cells, thereby modulating the insulin signaling pathway. PKR, facilitated by other protein kinases like JNK and IKK, heightens IRS1 phosphorylation at Ser312 and inhibits its tyrosine phosphorylation [64,65]. IκB kinase β (IKK-β) is a key mediator of inflammatory signaling pathways, particularly the NF-κB (nuclear factor kappa-light-chain-enhancer of activated B cells) pathway. In conditions of chronic inflammation, such as obesity, IKKβ is activated by pro-inflammatory cytokines like TNF-α (tumor necrosis factor-alpha) and IL-6 (interleukin-6). Activated IKKβ phosphorylates IRS proteins on serine residues, leading to their degradation and impairing insulin signaling. Additionally, IKKβ activation promotes the expression of inflammatory genes that further exacerbate insulin resistance [66]. JNK (c-Jun N-terminal Kinase) is a stress-activated protein kinase that is activated by various stressors, including inflammatory cytokines, oxidative stress, and lipid intermediates [67]. Once activated, JNK phosphorylates IRS proteins on serine residues, interfering with their ability to transmit insulin signals [68]. JNK activation also promotes the expression of pro-inflammatory genes and induces apoptotic pathways [69], contributing to insulin resistance and pancreatic β-cells dysfunction.

Although the exact location of the defect within the insulin signaling pathway remains uncertain, numerous key components were identified. These components encompass proximal elements such as insulin receptors, insulin receptor substrates, PI3K, and AKT/PKB, as well as distal elements representing various components downstream of AKT/PKB, including TBC1D4, GSK3, and PDE3B. Insulin resistance stems from malfunctions in one or more of these signaling components [70].

### 3.2. Inflammation and Oxidative Stress

Chronic low-grade inflammation and oxidative stress are associated with insulin resistance. Chronic low-grade inflammation, characterized by elevated levels of pro-inflammatory cytokines such as TNF-α, IL-6, and IL-1β, is a hallmark of obesity and insulin resistance [71,72]. Adipose tissue, particularly visceral adipose tissue, serves as a major source of pro-inflammatory cytokines in obesity [73]. These cytokines activate inflammatory signaling pathways, such as the NF-κB pathway, in target tissues including skeletal muscle, liver, and adipose tissue. Activation of NF-κB leads to increased expression of inflammatory genes, including those encoding cytokines, chemokines, and adhesion molecules [74]. Inflammatory signaling pathways can directly interfere with insulin signaling by promoting serine phosphorylation of IRS proteins, impairing their ability to transmit insulin signals, as described earlier. Additionally, inflammation contributes to insulin resistance by promoting adipose tissue dysfunction, ectopic lipid accumulation, and systemic insulin resistance.

Oxidative stress arises from an imbalance between the production of reactive oxygen species (ROS) and the ability of antioxidant defense mechanisms to neutralize them [75]. ROS, including superoxide radicals, hydrogen peroxide, and hydroxyl radicals, are generated by various cellular processes, including mitochondrial respiration, NADPH oxidase activity, and enzymatic reactions [76]. In conditions of excess nutrient intake, mitochondrial dysfunction and increased flux through metabolic pathways, such as the electron transport chain and the pentose phosphate pathway, can lead to increased ROS production. Elevated ROS levels can damage cellular macromolecules, including lipids, proteins, and DNA, leading to cellular dysfunction and death [77]. ROS can also directly impair insulin signaling by inhibiting insulin receptor autophosphorylation and activating serine kinases such as JNK and IKKβ, which phosphorylate IRS proteins and interfere with insulin signaling [78]. Additionally, ROS promote the production of pro-inflammatory cytokines and chemokines, exacerbating inflammation and insulin resistance [79].

### 3.3. Lipid Accumulation and Lipotoxicity

Lipid accumulation and lipotoxicity are key factors contributing to the development of insulin resistance and metabolic dysfunction. Lipids, including triglycerides, cholesterol, and free fatty acids (FFAs), play essential roles as energy substrates and signaling molecules in cellular metabolism [80]. However, excessive lipid accumulation, particularly in non-adipose tissues such as skeletal muscle, liver, and pancreatic β-cells, can lead to cellular dysfunction and insulin resistance [81]. Lipid accumulation and lipotoxicity contribute to insulin resistance in several ways.

Ectopic lipid accumulation refers to the deposition of lipids, such as triglycerides and fatty acids, in tissues where they are not typically stored, such as skeletal muscle, liver, and pancreatic β-cells [82]. While adipose tissue serves as the primary site for lipid storage, excess lipid uptake or impaired lipid metabolism can lead to lipid spillover into non-adipose tissues, contributing to various metabolic dysfunctions [83]. Skeletal muscle is a major site for glucose uptake and utilization, making it crucial for maintaining glucose homeostasis [84]. In conditions of positive energy balance or impaired lipid metabolism, skeletal muscle can accumulate excess lipids, particularly intramuscular triglycerides (IMTGs) and fatty acid metabolites [85]. Ectopic lipid accumulation in skeletal muscle is strongly associated with insulin resistance, as it disrupts insulin signaling pathways and impairs glucose uptake and utilization. Lipid metabolites, such as diacylglycerol (DAG) and ceramides, can activate serine kinases, including protein kinase C (PKC) isoforms and JNK, leading to the phosphorylation of IRS and inhibition of insulin signaling [4,86,87]. The liver plays a central role in lipid metabolism, including fatty acid uptake, synthesis, oxidation, and export. Excessive dietary fat intake, increased lipolysis from adipose tissue, or impaired hepatic lipid metabolism can lead to hepatic lipid accumulation, a condition known as non-alcoholic fatty liver disease (NAFLD) [88]. Hepatic lipid accumulation is strongly associated with insulin resistance and type 2 diabetes, as it disrupts hepatic insulin signaling and promotes gluconeogenesis, leading to increased hepatic glucose production [89]. Additionally, the accumulation of lipid intermediates, such as ceramides and DAG, can impair insulin signaling and promote hepatic inflammation and fibrosis [90]. Ectopic lipid accumulation in pancreatic β-cells has emerged as a potential contributor to β-cell dysfunction and insulin resistance. Excess lipid exposure can impair β-cell function by inducing lipotoxicity, oxidative stress, and ER stress, leading to impaired insulin secretion and glucose intolerance. Lipid metabolites, such as ceramides, were shown to inhibit insulin signaling pathways and promote β-cell apoptosis, contributing to the progression of type 2 diabetes [2]. Dysfunction of adipose tissue, characterized by adipocyte hypertrophy, inflammation, and altered adipokine secretion, can promote ectopic lipid accumulation in non-adipose tissues [74]. Enlarged adipocytes are less insulin-sensitive and exhibit increased lipolysis, releasing more fatty acids into circulation [91]. These excess fatty acids can then be taken up by non-adipose tissues, exacerbating ectopic lipid accumulation and insulin resistance.

### 3.4. Mitochondrial Dysfunction

Mitochondria serve as the cellular power stations, playing vital roles in fundamental cellular functions such as generating ATP, regulating intracellular calcium levels, producing and detoxifying reactive oxygen species, modulating apoptotic cell death, and activating caspase proteases [92]. Their dysfunction refers to impaired function of the mitochondria, the organelles responsible for cellular energy production through oxidative phosphorylation (OXPHOS) and various metabolic processes. Emerging evidence suggests that mitochondrial dysfunction plays a crucial role in the development of insulin resistance and metabolic disorders [93].

Mitochondrial dysfunction can lead to impaired oxidative phosphorylation, resulting in decreased ATP production and energy deficits in cells. Reduced ATP levels can impair cellular processes that require energy, including insulin-stimulated glucose uptake and metabolism. Skeletal muscle, which is a major site of glucose disposal, is particularly sensitive to mitochondrial dysfunction. Impaired mitochondrial function in skeletal muscle can lead to decreased mitochondrial ATP production, reduced glucose oxidation, and accumulation of lipid intermediates, contributing to insulin resistance [94].

Mitochondria are a major source of reactive oxygen species (ROS), which are natural byproducts of mitochondrial respiration. Under conditions of mitochondrial dysfunction, excessive ROS production can occur, leading to oxidative stress and damage to cellular macromolecules, including lipids, proteins, and DNA. ROS can also impair insulin signaling pathways by directly modifying IRS and inhibiting insulin-stimulated glucose uptake. Moreover, ROS-mediated oxidative stress can activate stress-sensitive kinases, such as JNK and IKK, which can phosphorylate IRS proteins on serine residues and interfere with insulin signaling [77,78].

Mitochondria undergo dynamic processes of fusion, fission, and mitophagy to maintain mitochondrial function and integrity [95,96]. Dysregulation of mitochondrial dynamics and impaired mitophagy can lead to the accumulation of dysfunctional mitochondria and mitochondrial DNA (mtDNA) damage. Accumulation of damaged mitochondria can further exacerbate oxidative stress and impair cellular function, contributing to insulin resistance. Additionally, impaired mitochondrial quality control mechanisms were implicated in the pathogenesis of metabolic disorders such as type 2 diabetes and obesity [92].

Mitochondrial dysfunction can also disrupt lipid metabolism, leading to ectopic lipid accumulation and lipotoxicity in insulin-sensitive tissues. Impaired mitochondrial β-oxidation of fatty acids can lead to increased accumulation of lipid intermediates, such as diacylglycerol (DAG) and ceramides, which can impair insulin signaling pathways and contribute to insulin resistance. Moreover, dysfunctional mitochondria may exhibit altered substrate utilization and inefficient energy metabolism, further exacerbating metabolic dysfunction and insulin resistance [94].

## 4. Physical Activity and Insulin Resistance

Engaging in physical exercise encompasses activities that are systematically planned, structured, and undertaken to enhance or sustain physical fitness and overall health [97]. The advantages of physical exercise are extensive, impacting both physical and mental well-being [98]. Regular participation in physical exercise significantly enhances cardiovascular health, lowering the risk of heart disease, stroke, and hypertension. Additionally, it boosts bone density, thereby diminishing the likelihood of osteoporosis and fractures. Maintaining an active lifestyle aids in weight management, enhances insulin sensitivity, and decreases the risk of type 2 diabetes [99,100,101,102].

Physical exercise comes in many forms, including aerobic activities, strength training, and flexibility exercises. Aerobic activities, such as running, biking, and swimming, are highly effective for enhancing cardiovascular health and burning calories. Strength training, which includes exercises like weight lifting and resistance band routines, builds muscle strength and preserves bone density. Flexibility exercises, like yoga and stretching, enhance mobility and reduce injury risk. Exercise intensity and duration can differ greatly from person to person, making it challenging to assess the overall effectiveness of exercise programs.

Epidemiological studies have consistently demonstrated a strong association between physical activity levels and insulin sensitivity, highlighting the crucial role of exercise in metabolic health [103]. Regular exercise is widely recognized for its beneficial effects on metabolic health, particularly in addressing obesity and enhancing insulin sensitivity. A meta-analysis was conducted to evaluate the efficacy of structured exercise intervention programs for insulin resistance in T2DM. The findings underscore the effectiveness of regular exercise in improving glycemic control, thus warranting its recommendation as a therapeutic strategy for reducing IR, supported by a moderate level of evidence [104]. Studies have shown that physical activity with high energy expenditure and higher intensity has greater benefits for increasing insulin sensitivity. This includes high-intensity interval training (HIIT) [105].

Currently, the homeostasis model assessment of insulin resistance index, or HOMA-IR, is most commonly used to diagnose insulin resistance. It involves assessing the disruption of the homeostatic relationship between plasma insulin and glucose concentrations [106]. It was shown that during aerobic exercise, the intensity of which is gradually increased, a reduction in abdominal fat and subcutaneous fat is observed after 6 months. These changes are also believed to cause a 16% decrease in the HOMA-IR index [107]. However, a similar study using exercise training in which participants reduced visceral fat area after 12 weeks showed no effect on the HOMA-IR index [108]. However, it should be taken into account that this study lasted twice as long despite being conducted on a similar group. In addition, the authors emphasize that the HOMA-IR index does not take into account small changes within the body that can significantly affect changes in insulin sensitivity [109].

Strong evidence suggests that physical inactivity is closely linked to islet cell insufficiency. Adopting a sedentary lifestyle can exacerbate insulin resistance by imposing a greater workload on islets and diminishing their efficiency. This phenomenon occurs through various pathways, including ER stress, mitochondrial dysfunction, oxidative stress, and inflammation, ultimately promoting β-cell apoptosis and death [110]. Conversely, engaging in physical training and exercise serves to preserve and even restore islet function, thereby enhancing peripheral insulin sensitivity [111]. Furthermore, exercise can stimulate β-cell proliferation by increasing circulating levels of growth factors such as growth hormone, insulin-like growth factor 1 (IGF-1), and glucagon-like peptide 1 (GLP-1) [112]. Inactive individuals were found to exhibit lower β-cell sufficiency compared to trained individuals. Long periods of inactivity lead to increased fasting plasma glucose levels, while moderate-to-high-intensity exercise can reverse these changes, improving islet function and glucose metabolism [113]. Sedentary individuals also show reduced insulin sensitivity and β-cell sufficiency in response to carbohydrate intake [111]. Moreover, individuals with lower physical activity levels, including T2DM patients, exhibit compromised islet function and irregular glucose metabolism compared to those engaged in aerobic exercise [114]. Short-term exercise interventions were shown to improve pancreatic β-cell activity and glucose metabolism [115]. Additionally, studies in animal models have demonstrated that inactive obese diabetic rats have lower pancreatic β-cell function compared to exercise groups [116,117]. These findings collectively emphasize the importance of physical activity in preserving and enhancing islet function while underscoring the detrimental effects of physical inactivity on β-cell health.

Physical inactivity was linked to disruptions in the expression, translocation, and function of genes and proteins involved in glucose homeostasis, while aerobic exercise was shown to stimulate these genes [10,118,119,120]. GLUT4, crucial for insulin-stimulated glucose uptake, is particularly affected. Research indicates that physical inactivity can lead to decreased *GLUT4* expression in skeletal muscles, contributing to insulin resistance. Studies using the hyperinsulinemic–euglycemic clamp method have demonstrated that prolonged bed rest significantly down-regulated genes related to insulin signaling, including *GLUT4*, *HK2* (hexokinase 2), *RRAD* (Ras-related glycolysis inhibitor and calcium channel regulator), and *TXNIP*, thereby reducing insulin sensitivity in skeletal muscles [119]. Conversely, evidence suggests that physical activity increases the expression of *IRS1*, *IRS2*, *Akt*, *PI3 kinase*, and *GLUT4* in animal models [121]. Similarly, studies in humans have shown that short-term bed rest interventions result in downregulation of GLUT4, HK2, GS, and Akt proteins, leading to decreased insulin sensitivity in skeletal muscles [118]. These findings underscore the importance of regular physical activity in maintaining proper gene expression and insulin sensitivity in skeletal muscles.

As was pointed out, inflammation may play a role in insulin resistance. However, in the context of skeletal muscle, no macrophages, lymphocytes or granulocytes were observed in myocytes in images obtained by electron microscopy [122]. For this reason, inflammation in skeletal muscle is thought to be caused by the presence of immune cells in perimuscular adipose tissue [123]. It is the inflammation of adipose tissue that is increasingly recognized as an activating factor in the development of IR. However, despite the clear contribution of adipose tissue to the development of the disorder, there are still few reports on the effect of physical activity on it [12]. There are many studies confirming the effect of physical activity on plasma levels of specific pro- and anti-inflammatory cytokines [124]. Studies indicate that a sedentary lifestyle can lead to increased inflammatory markers. Replacing sedentary activities with physical activity resulted in a reduction in circulating cytokines and an improvement in insulin sensitivity among obese adults [125]. Additional evidence suggested that even short periods of physical inactivity in healthy individuals could trigger inflammatory responses and increase the risk of insulin resistance and type 2 diabetes mellitus [126]. Regular physical exercise is consistently linked to alterations in inflammatory cytokine profiles, favoring a shift towards anti-inflammatory responses. Notably, exercise was shown to elevate levels of anti-inflammatory cytokines while reducing pro-inflammatory ones, such as TNF-α and IL-1β, which are implicated in the pathogenesis of diabetes [127,128]. Furthermore, studies have demonstrated that exercise interventions, particularly in combination with dietary modifications, lead to reductions in circulating levels of inflammatory markers like IL-6, IL-18, and CRP (C-reactive protein), coupled with increased levels of adiponectin, a hormone with potent anti-inflammatory and insulin-sensitizing effects [129,130]. Additionally, physically active individuals tend to exhibit lower levels of leptin, a hormone associated with inflammation [131]. Importantly, exercise exerts anti-inflammatory effects by improving endothelial function and reducing peripheral markers of endothelial dysfunction, including soluble intracellular and vascular adhesion molecules, as well as granulocyte-macrophage colony-stimulating factor [132]. Given the undeniable contribution of adipose tissue to the development of IR, adipokines, which are signaling molecules produced and released by adipose tissue, also deserve special attention. Studies have shown that prolonged exercise affects the expression of leptin and adiponectin in subcutaneous adipose tissue in people at risk of developing T2DM. Compared to a control group containing healthy subjects, the expression of these adipokines normalized [133].

There is compelling evidence suggesting that physical inactivity contributes to increased oxidative stress levels [134]. Studies have indicated that individuals with sedentary lifestyles exhibit higher levels of oxidative damage markers, such as elevated malondialdehyde (MDA) and reduced total antioxidant capacity (TAC) in plasma, compared to their physically active counterparts [135]. Additionally, research has shown that inactive older individuals have diminished activity of key antioxidant enzymes like superoxide dismutase (SOD), catalase (CAT), and glutathione peroxidase (GPx), along with higher plasma MDA levels [136]. Interestingly, acute bouts of exercise were observed to transiently elevate reactive oxygen and nitrogen species (RONS) levels; however, this phenomenon serves as a stimulus for the up-regulation of endogenous antioxidant defenses [137]. Indeed, studies in untrained animals have demonstrated increased oxidant levels following acute exercise [138,139], yet long-term exercise training counteracts this effect by promoting the expression of antioxidant enzymes, consequently reducing free radical production. Further evidence from animal studies has revealed that endurance training leads to elevated levels of antioxidants and antioxidant enzymes in skeletal and cardiac muscles, thereby providing protection against oxidative stress [140,141,142].

There is compelling evidence suggesting that physical inactivity contributes to increased ceramide production [143]. Studies have shown that prolonged periods of inactivity lead to impaired insulin sensitivity by disrupting the trafficking and metabolism of lipids, including ceramides, in both cellular and plasma compartments. For instance, research has demonstrated that just two months of bed rest can result in the accumulation of saturated fats and sphingosine in muscle cells, ultimately impairing insulin sensitivity [144]. Similarly, findings from animal studies indicate that 14 days of inactivity can elevate ceramide levels, disrupt insulin signaling in skeletal muscle, and worsen glucose tolerance [145]. Moreover, direct evidence from studies on obese volunteers has linked increased muscle ceramide levels during physical inactivity to insulin resistance. Conversely, acute exercise was shown to reduce sphingolipid synthesis during recovery periods, leading to improved insulin sensitivity in trained individuals [146]. However, conflicting reports on the short-term effects of inactivity on ceramide levels and glucose homeostasis suggest that ceramides may require more prolonged exposure to exert pathogenic effects and disrupt insulin signaling [147].

Considerable evidence indicates that physical inactivity and sedentary behaviors have adverse effects on mitochondrial function [148,149]. Trained individuals typically demonstrate superior mitochondrial performance capacity compared to their sedentary counterparts [50]. For example, prolonged periods of inactivity were shown to impair mitochondrial respiratory function in skeletal muscles, leading to decreased mitochondrial oxidative capacity due to oxidative damage in immobilized tissues [149]. Furthermore, inactive subjects tend to exhibit lower mitochondrial oxidative capacity compared to their physically active counterparts. Active older adults, in particular, often display improved mitochondrial capacity, highlighting mitochondria as a critical therapeutic target for sedentary-related complications and insulin resistance [148]. Clinical evidence has also linked physical inactivity-induced insulin resistance to significant changes in mitochondrial genes involved in glucose metabolism [119]. A link between obesity and mitochondrial dysfunction was also observed. Expression of the mitochondrial gene *ESR1* in adipocytes, which is responsible for encoding estrogen receptor α (ERα), may be dependent on obesity [150]. Studies in animal models have shown that deletion of ERα from adipocytes increases their size and contributes to obesity [151]. However, long-term physical activity improves ERα function and increases the number of mitochondria in adipose tissue which then reduces insulin resistance [152].

A summary of the effect of physical activities on insulin resistance is shown in Table 1.

It is also important in this review to highlight gender differences that may affect insulin resistance. A woman’s body is hormonally and biologically different from a man’s, which also contributes to a different response to physical activity. One such difference is the presence of the female sex hormone, estrogen. It was suggested that estrogen protects against the development of insulin resistance through two mechanisms: modulating the metabolic processes involved in energy balance and down-regulating and/or repressing inflammation [153]. For this reason, men are thought to be more susceptible to metabolic syndromes. However, the situation is different for post-menopausal women, as with decreasing estrogen levels, their risk of developing IR increases. Studies have also proven that menopause is a factor that increases the risk of weight gain and impairs glucose tolerance [154,155]. On the other hand, studies have proven that the incidence of metabolic syndrome and diabetes is closely related to testosterone and changes in abdominal body composition [156]. It was proven that low testosterone levels are strongly correlated with the incidence of insulin resistance and type 2 diabetes [157]. Also common is high visceral obesity with low testosterone levels. It was suggested that changes in abdominal body composition are a secondary consequence of metabolic syndrome [158]. In a study examining gender differences in the effect of type 2 diabetes on exercise performance, a decrease in peak oxygen consumption was observed in diabetic men and women compared to controls. However, it should be noted that this abnormality was significantly greater in the female group (24%) than in the male group (16%) [159]. Another study comparing the effects of physical activity on IR in both men and women with type 2 diabetes involved a 12-week training program consisting of aerobic and resistance training three days a week. The men showed increased improvements in peak oxygen consumption and stroke volume index compared to the women’s group. However, only women significantly improved heart rate recovery (after 1 min, 48%), insulin resistance (25%), insulin C-peptide (27%) and right ventricular systolic function (21%) [160]. Another study looked at the relationship between participation in strength training and insulin resistance. This was a cross-sectional study using data from the National Health and Nutrition Examination Survey. The study found no significant association between strength training and insulin resistance in women. However, higher HOMA-IR was observed in men in the absence of strength training compared to men who reported moderate to high levels of strength training. From the data, it was also estimated that men who did not strength train had a 2.42 times higher risk of insulin resistance compared to men who exercised at a moderate level and a 2.5 times higher risk compared to men who strength trained intensively [161].

## 5. Conclusions

Studies consistently highlight the association between physical activity levels and insulin sensitivity, underscoring exercise’s pivotal role in metabolic health. Regular exercise is widely acknowledged for its positive effects on metabolic parameters, particularly in addressing obesity and enhancing insulin sensitivity. A meta-analysis evaluating structured exercise intervention programs for insulin resistance in T2DM reinforced the effectiveness of regular exercise in improving glycemic control, substantiating its recommendation as a therapeutic approach for reducing insulin resistance. One of the key pathways through which exercise exerts its beneficial effects is by modulating ceramide production. Ceramides, lipid molecules associated with insulin resistance, are influenced by physical activity levels. Regular exercise was shown to reduce ceramide levels, thereby improving cellular lipid metabolism and insulin sensitivity.

Mitochondrial dysfunction is another target of physical activity interventions aimed at improving insulin sensitivity. Sedentary behaviors are associated with impaired mitochondrial function, leading to decreased oxidative capacity and tissue damage. In contrast, regular exercise promotes mitochondrial biogenesis and enhances mitochondrial function, thereby enhancing cellular energy production and metabolic efficiency. By improving mitochondrial function, exercise helps alleviate oxidative stress and mitigate insulin resistance.

Furthermore, physical activity has anti-inflammatory effects that can counteract the chronic low-grade inflammation observed in individuals with insulin resistance. Sedentary lifestyles are associated with elevated levels of inflammatory cytokines, which contribute to the development of metabolic disorders. Regular exercise was shown to reduce systemic inflammation by decreasing circulating levels of pro-inflammatory cytokines and increasing anti-inflammatory cytokines. By modulating inflammatory pathways, exercise helps improve insulin sensitivity and metabolic health.

In addition to its anti-inflammatory effects, physical activity positively influences pancreatic beta cell function and insulin secretion. Sedentary behaviors increase the workload on pancreatic islets, leading to impaired insulin secretion and reduced efficiency in glucose metabolism. Conversely, regular exercise preserves and enhances β-cell function, promoting insulin secretion and glucose homeostasis. Aerobic exercise, resistance training, and high-intensity interval training have all been shown to improve beta cell function and insulin sensitivity.

Overall, physical activity has multifaceted effects on insulin resistance, targeting various mechanisms involved in metabolic dysfunction. By reducing ceramide production, improving mitochondrial function, and modulating inflammatory pathways, exercise helps enhance insulin sensitivity and promote metabolic health. Additionally, exercise promotes β-cell function and insulin secretion, further contributing to glucose homeostasis. Furthermore, physical inactivity disrupts gene and protein expression involved in glucose homeostasis, leading to decreased insulin sensitivity. In contrast, aerobic exercise up-regulates key genes related to insulin signaling, such as *GLUT4*, promoting insulin-stimulated glucose uptake and improving insulin sensitivity in skeletal muscles. Physical inactivity also contributes to elevated oxidative stress levels, characterized by increased oxidative damage markers and diminished antioxidant enzyme activity. In contrast, regular exercise enhances antioxidant defenses, reducing free radical production and mitigating oxidative stress-induced insulin resistance.

Encouraging individuals to adopt active lifestyles and engage in regular exercise is essential for preventing and managing insulin resistance and related metabolic disorders. Public health initiatives aimed at promoting physical activity can have significant benefits for improving metabolic health and reducing the burden of insulin resistance and type 2 diabetes. By addressing the underlying mechanisms of insulin resistance, physical activity interventions offer promising strategies for improving overall health and well-being.

## Figures and Tables

**Table 1:** Effect of physical activities on insulin resistance.

Mechanism	Research Group	Physical Activity	Impact of Physical Activity	References
Islet cells	12 type 2 diabetic male patients	12-week programme on ergometer cycle (5 sessions/week)	preserve and even restore islet function, thereby enhancing peripheral insulin sensitivity	[111]
	40 type 2 diabetic patients	accumulated million steps for 1 year	even short-term exercise interventions improve pancreatic β-cell activity and glucose metabolism	[113]
Genes and proteins	female Wistar rats	two 3 h exercise bouts, separated by one 45 min rest period	increased expression of IRS-1, IRS-2, Akt, PI3 kinase, and Glut-4	[121]
Inflammation	General data from review articles. A detailed summary was presented in a previous paper [124]		increased levels of anti-inflammatory cytokines and reduced pro-inflammatory ones	[124,127,128]
	100 healthy volunteers (48 men, and 52 women)	lifestyle evaluation	decreased level of leptin	[131]
Oxidative Stress	75 type 2 diabetic patients	based on Global Physical Activity Questionnaire	lower levels of oxidative damage markers, such as MDA and higher TAC in plasma	[135]
	87 older males	engage in any physical activity every day for 12 months	higher activity of key antioxidant enzymes like SOD, CAT and GPx	[136]
	344 male rats	10 weeks of exercise training	endurance training leads to elevated levels of antioxidants and antioxidant enzymes in skeletal and cardiac muscles	[140,141,142]
Lipid Accumulation and Lipotoxicity	14 obese sedentary individuals, 15 type 2 diabetic patients and 15 endurance-trained athletes	ergometer cycle for 1.5 h	acute exercise reduces sphingolipid synthesis during recovery periods, leading to improved insulin sensitivity in trained individuals	[146]
Mitochondrial Dysfunction	26 sedentary (<1 exercise session/week) men	12-week intensive exercise intervention	increased number of mitochondria in adipose tissue	[152]

## Data Availability

Not applicable.
